# PLEX.I: a tool to discover features in multiplex networks that reflect clinical variation

**DOI:** 10.3389/fgene.2023.1274637

**Published:** 2023-10-19

**Authors:** Behnam Yousefi, Farzaneh Firoozbakht, Federico Melograna, Benno Schwikowski, Kristel Van Steen

**Affiliations:** ^1^ Computational Systems Biomedicine Lab, Institut Pasteur, Université Paris Cité, Paris, France; ^2^ École Doctorale Complexite du Vivant, Sorbonne Université, Paris, France; ^3^ BIO3—Laboratory for Systems Medicine, KU Leuven, Leuven, Belgium; ^4^ Institute for Computational Systems Biology, University of Hamburg, Hamburg, Germany; ^5^ BIO3—Laboratory for Systems Genetics, GIGA-R Medical Genomics, University of Liège, Liège, Belgium

**Keywords:** biological interaction networks, functional genomics, gene regulation, machine learning, software

## Abstract

Molecular profiling technologies, such as RNA sequencing, offer new opportunities to better discover and understand the molecular networks involved in complex biological processes. Clinically important variations of diseases, or responses to treatment, are often reflected, or even caused, by the dysregulation of molecular interaction networks specific to particular network regions. In this work, we propose the *R* package PLEX.I, that allows quantifying and testing variation in the direct neighborhood of a given node between networks corresponding to different conditions or states. We illustrate PLEX.I in two applications in which we discover variation that is associated with different responses to tamoxifen treatment and to sex-specific responses to bacterial stimuli. In the first case, PLEX.I analysis identifies two known pathways i) that have already been implicated in the same context as the tamoxifen mechanism of action, and ii) that would have not have been identified using classical differential gene expression analysis.

## 1 Introduction

Interactions between different biological entities are crucial for the function of biological organisms. In biological networks, nodes can represent genes, proteins or microbes, and their interactions can be defined by edges, which can be either binary or weighted (Loscalzo, 2017). Gene co-expression networks (GCNs), for instance, are biological networks whose nodes represent genes and whose edges between them are weighted by a measure of their co-expression, such as correlation. Highly connected sets of genes are often found to be involved in the same functional context.

*Multiplex networks* consist of multiple sets of edges between the same set of nodes (Hammoud and Kramer, 2020). Each edge set, referred to as a layer, can represent a distinct type of data (e.g., transcriptomics and proteomics) or clinical condition (e.g., disease/health and responder/non-responder to a drug). Various methods have been proposed for the analysis of multiplex networks, many of which detect those nodes whose set of neighbors (*neighborhood*) is highly consistent across layers (Buphamalai et al., 2021; Mahapatra et al., 2021; Peng et al., 2021). With highly consistent neighborhoods across layers may be prioritized as the most interesting ones for downstream computational or biological analysis. This type of method is often used to aggregate networks from different omics datasets.

Other methods identify nodes whose neighborhoods vary from one layer to the other. These methods are, in particular, useful for the detection of dysregulated genes that can be associated with different clinical conditions such as diseases and specific responses to treatments (Loscalzo, 2017). To quantify the variations in the local neighborhood of each node (e.g., gene) between two given conditions (or clinical states), such as case-control or drug sensitive-resistant status, several methods have been used in the literature, which have been reviewed in Lichtblau et al. (2016), van Dam et al. (2018), and Bródka et al. (2018). These methods are essentially based on comparing a measure of degree centrality, such as in Fuller et al. (2007) and de la Fuente (2010) between the two layers. However, such measures can fail in recognizing neighborhood variation if the node centrality remains unchanged. To overcome this shortage, we propose a tool named PLEX.I to discover local variations of such networks, and to test for statistical significance of such variation.

The input to PLEX.I is a multiplex network. In Scenario I, we consider a two-layer network, where each layer corresponds to a condition; and, the aim is to detect nodes whose neighborhood significantly changes from one condition to the other. In Scenario II, we consider a set of two-layer networks, each for one individual; and, the aim is to detect nodes whose neighborhood variation, from one layer to the other, is associated with a particular phenotype across individuals. We have shown an application of both scenarios on human gut microbiomes data in our previous study (Yousefi et al., 2023).

Here, we illustrate the potential of PLEX.I in Scenario I and II in broader applications. In particular, for Scenario I, we detect genes whose regulatory neighbors are associated with drug response on human cancer cell lines. And, for Scenario II, we detect genes whose neighborhood variation is associated with sex (see Usage Examples).

## 2 Methods and implementation

**FIGURE 1:**
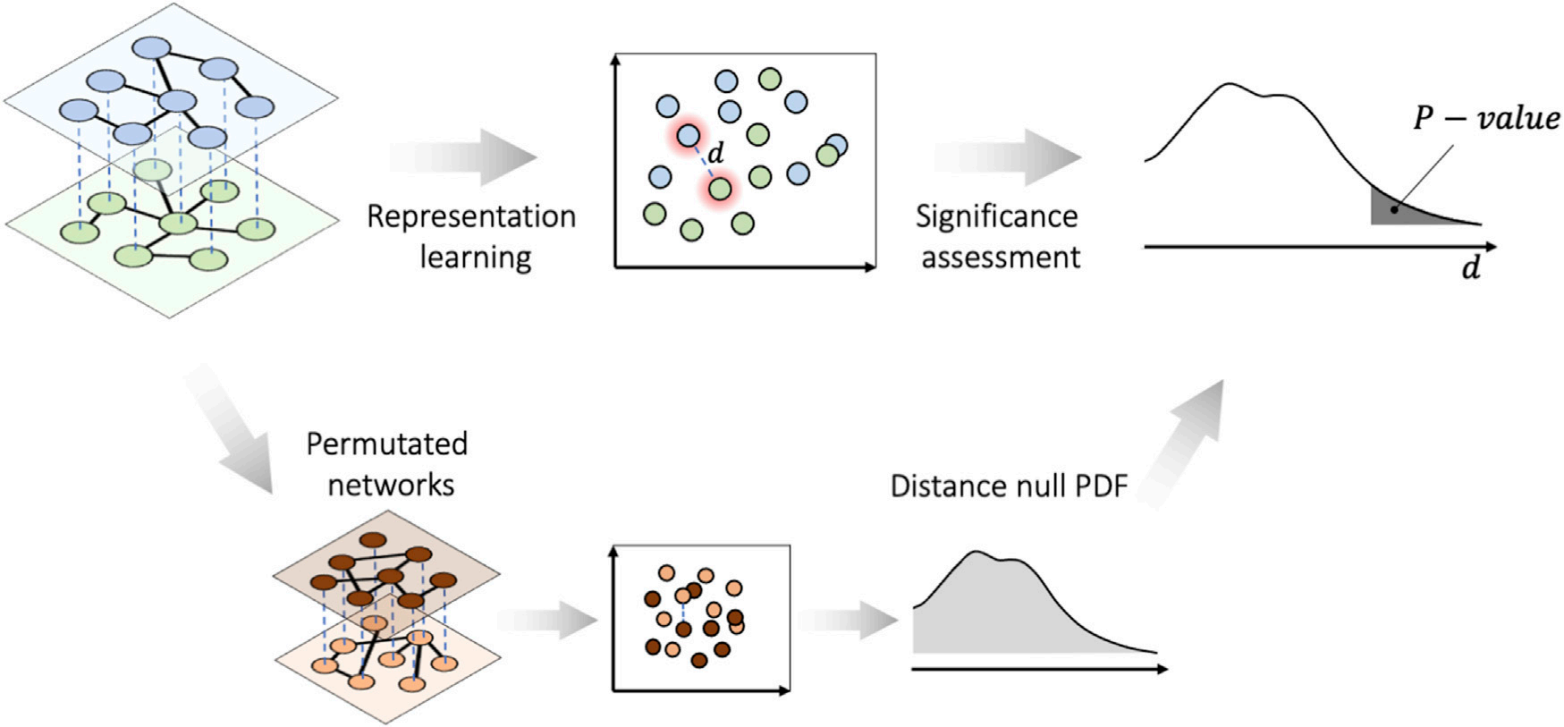
PLEX.I essentials for Scenario I. The nodes in all layers of the multiplex network, along with the permuted networks, are embedded into a vector space. Then the significance of the distances is assessed on the basis of the probability distribution function (PDF) of the null distances.

Considering a two-layer multiplex network as the input, in the first phase, PLEX.I performs *representation learning* that constructs a map, which, for each layer, projects each node onto a point in a low-dimensional *embedding space* such that the pairwise graph distances in each layer are approximated by the pairwise distances of the corresponding two points in the embedding space. As the pairwise graph distances also reflect local variations in the neighborhood of any given node between two layers, the distance d in the embedding space can also be interpreted as a measure of the original variation in the node neighborhood between the two layers. In the second phase, PLEX.I performs an assessment of statistical significance for each gene, as follows. In Scenario I, for the (gene-specific) distance d , a *p*-value is computed by comparing d against a background distribution generated from permuted versions of the multiplex network (Figure 1). In Scenario II, a (again, gene-specific) distance $d_{i}$ is calculated for each individual i and a *p*-value is computed that reflects the association of this distance with a phenotype of interest. The details of both phases are described in the following.

### 2.1 Phase I. Representation learning

Given a multiplex network with *n* nodes, PLEX.I first maps the nodes of each layer to a common embedding space of dimension k (a user-defined parameter). To obtain a simultaneous embedding of all network layers, we designed an encoder-decoder neural network (EDNN) with n -dimensional inputs and outputs, and a k -dimensional bottleneck layer (Ietswaart et al., 2021; Yousefi et al., 2023). For a discussion of the suitability of this approach, for our application, over other network representation learning methods, we refer to Hamilton et al. (2017), Yousefi et al. (2023) *.* For each given node v of each layer, the input of the EDNN is the vector of edge weights between v and any node w of the layer. The output the EDNN is trained to generate is either the input edge weights or the vector of empirical probabilities to reach w after a fixed-length random walk (Yousefi et al., 2023), which can be selected by the user.

The activation functions of the EDNN output layer are *logistic* functions, as the decoder outputs are probabilities, while activation functions of the bottleneck layer can be chosen by the user. The user can also opt for different distance measures; we set the *cosine distance* as default as it performs best in our previous study (Yousefi et al., 2023). The code for the simulation study is described in the Supplementary Material S1.

### 2.2 Phase II. Significance assessment

Scenario I: To assess the statistical significance of a distance *d* in the embedding space, PLEX.I constructs an empirical distribution of distances *d* over a null model of multiplex networks. The user can choose from two possible background models: distances generated from i) random permutation of the (weighted) adjacency matrix, and ii) random replacement of edges that preserves the degree distribution.

Scenario II: To assess the statistical association of a distance *d* with a particular phenotype across individuals, different tests can be applied based on the nature of the phenotype. PLEX.I uses a Wilcoxon Rank-Sum test for the case of binary phenotypes and a correlation test (e.g., Kendall’s τ ) for continuous phenotypes.

The training of neural networks, with their non-convex cost functions, requires solving complex optimization problems. Training neural networks therefore requires an iterative optimization algorithm with random initialization. Different initializations, however, may result in different EDNNs, and, with that, in embedding spaces. To obtain robust results, we therefore train EDNN several times (the default number of repeat is 50), with different initializations. For each repeat, we calculate the distances and *p*-values; then aggregate all *p*-values into a single *p*-value using Fisher’s combined probability (Yi et al., 2018). Finally, PLEX.I implements different options to adjust the resulting n *p*-values for multiple testing (see also Supplementary Material S1).

### 2.3 Usage data

In Scenario I, we used the RNAseq gene expression data from the PRISM dataset (Corsello et al., 2020). We considered cell lines derived from lung cancer tumors with responses to Tamoxifen drug. We then calculated gene co-expression networks, for responders and non-responders, with 2,000 genes with the largest expression variance resulting in $\left(\begin{array}{l} 2000\\ 2 \end{array}\right)=1,999,000$ edges. As for the input multiplex network, one network layer was constructed from the transcriptomes of those cell lines that were found to respond to tamoxifen treatment (27 samples), the other was derived from those that did not (37 samples)—see Supplementary Material S2 for more details.

In Scenario II, we used transcriptome measurements for healthy human blood samples before and after bacterial stimulation (Thomas et al., 2015; Piasecka et al., 2018). We considered 370 samples with equal numbers of males and females. The data contains the NanoString expression of 564 genes resulting in $\left(\begin{array}{l} 564\\ 2 \end{array}\right)=158,766$ edges in each network. In the context of Scenario II, we show how PLEX.I can also be used to explore local neighborhood variations between individual specific networks (ISNs). ISNs model pairwise gene co-expression in individuals that are derived from single transcriptome measurements (Melograna et al., 2023). Here, we derived ISNs using the LIONESS method (Kujjer et al., 2019a; Kujjer et al., 2019b). We restricted our analysis to the three bacterial stimulation of *Escherichia coli*, *Staphylococcus aureus*, and *Staphylococcal enterotoxin B*. For each stimulation group and each individual, we constructed a multiplex network, one layer corresponding to the stimulation case and the other layer corresponding to the unstimulated case.

## 3 Results

**FIGURE 2:**
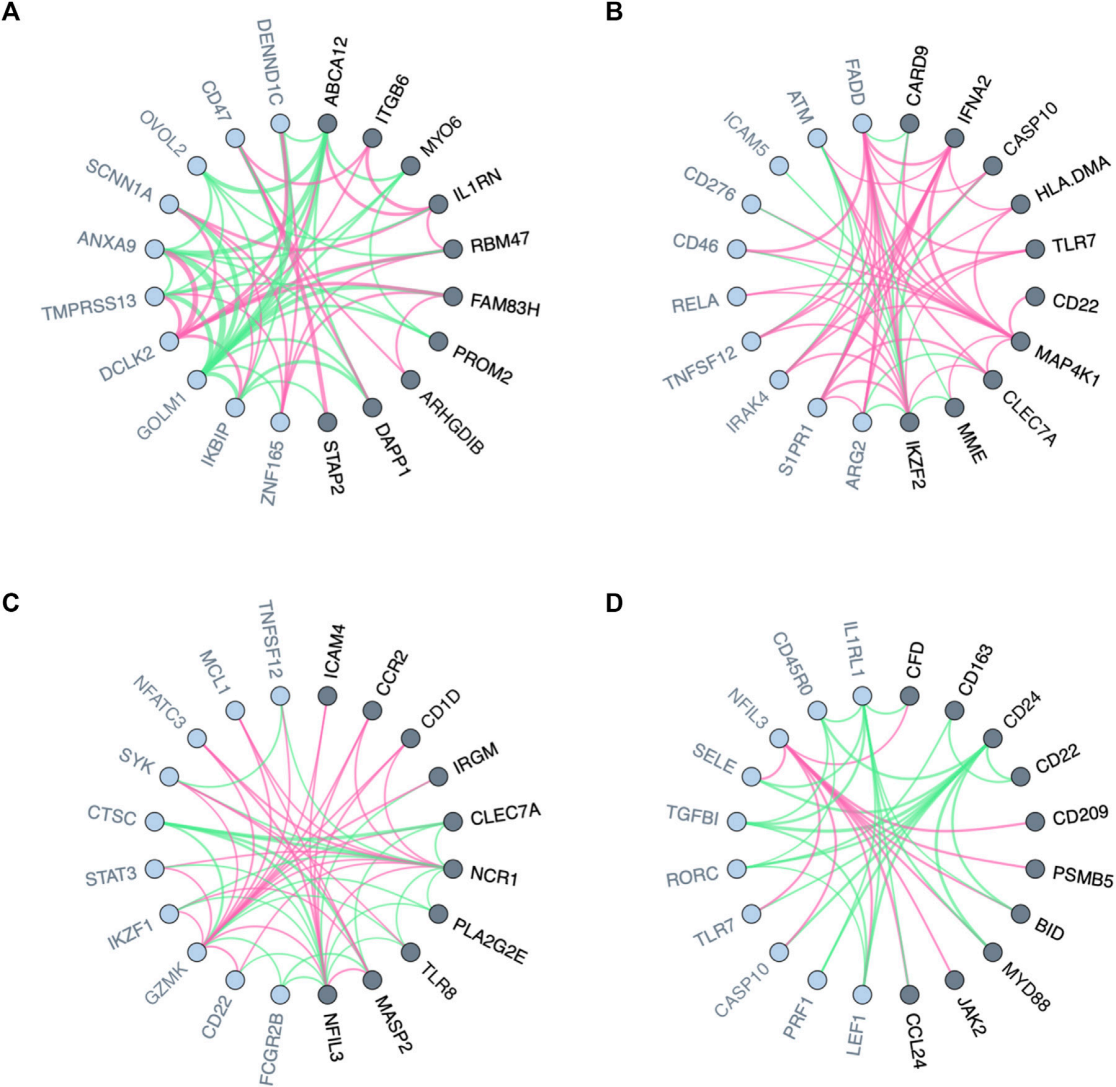
Visualized variation in the neighborhood of nodes in biological networks between two conditions or states. **(A)** Local variation in gene co-expression networks for responders and non-responders to Tamoxifen. **(B–D)** Local variation in interaction networks for the blood trascriptome of males and females stimulated, respectively, by *Escherichia coli*, *Staphylococcus aureus*, and Staphylococcal enterotoxin B. Genes (network nodes) detected by PLEX.I are highlighted in dark gray; their distance-1 neighbors are marked in light gray. The red and green edges show positive and negative edge connectivity differences, respectively, towards baseline [**(A)**: non-response; **(B–D)**: no stimulation].

Genes whose neighbors in GCN vary between populations with different clinical conditions appear to have a transcriptome regulatory effect, thus, are likely to explain the differences (Van Dam et al., 2018). In the context of personalized medicine, for instance, genes with differential co-expression patterns between drug responders and non-responders may play key roles in drug resistance. As a first usage example, we used PLEX.I to discover genes whose neighborhood variation is associated, in cell lines, with different responses to tamoxifen treatment. The input to PLEX.I consisted of gene co-expression networks, derived from cell lines of the PRISM dataset (Corsello et al., 2020). Using PLEX.I we identified 42 genes whose gene co-expression neighborhood varies significantly between the responsive and the non-responsive cell lines (Supplementary Table S1). Figure 2A shows the 10 most significant genes and their immediate neighbors. The edge weight shown represents the difference in the corresponding edge weights between the responder and non-responder networks. The results for an over representation analysis of these 42 genes, using the *Reactome* Analysis Tools–Analyse gene list (https://reactome.org), is shown in Supplementary Table S2. The two most strongly enriched pathways were *Interleukin* and *nuclear factor kappaB signaling*. Corroborating the PLEX.I analysis, these two pathways have already been found to interact specifically in the context of estrogen sensing, and, with that, in the context of the tamoxifen mechanism of action (Liu et al., 2005; Shao et al., 2015). We refer the reader to (Matariek et al., 2022; Howell and Howell, 2023) for an overview of the tamoxifen applications.

The interaction of the microbiome with the immune system has long been recognized to be of key importance for health and disease (Zheng et al., 2020). In the context of Scenario II, PLEX.I was applied on ISNs of blood transcriptomes stimulated with different microbes. For each microbial stimulation, we identified genes whose neighborhood variation is significantly associated with sex. Figures 2B–D shows the 10 most significant genes, respectively, for *Escherichia coli*, *Staphylococcus aureus*, and *Staphylococcal enterotoxin B* stimulations (see Supplementary Table S1). Similar to the previous use case, edge weight differences between aggregated networks across ISNs are shown as the edge colors.

It is worth mentioning that none of the genes identified by PLEX.I in either scenario were found to be differentially expressed between responders and non-responders or between unstimulated and stimulated groups (see Supplementary Material S2). For Scenario I, in particular, PLEX.I thus revealed plausible biological mechanisms underlying tamoxifen treatment that would not have been found using straightforward differential gene expression analysis, which considers only expression levels of individual genes. More details on the implementation of the usage examples with the implementation codes are provided in the Supplementary Material S1. The related analysis codes are freely available via the above GitHub repository.

## 4 Discussion

PLEX.I quantifies distances between the local neighborhoods of network nodes between two conditions or states and assesses their significance using a statistical test. The nodes of the network can represent any entity such as genes, microbes, and individuals. PLEX.I can therefore detect the variation in the neighborhood of any such network between two conditions or time points. In this manuscript we considered biological networks of gene regulations derived from a population of samples and biological ISNs, for Scenario I and Scenario II, respectively. Identified variations may highlight complementary information to commonly used node-oriented differential analysis strategies, as illustrated such as those proposed in Giri et al. (2023), Thomas et al. (2015), and Piasecka et al. (2018). A potential third scenario is to consider a set of two-layer networks, each for a pair of individuals, aiming at calculating similarities in local neighborhoods between pairs of individuals to cluster them. For future versions of PLEX.I, we plan additional features, such as the application on the multilayer networks in the third scenario mentioned above.

PLEX.I is freely available under a GPL-3 license on CRAN (https://cran.r-project.org/web/packages/PLEXI/index.html) and on GitHub (https://github.com/behnam-yousefi/PLEXI). It is written in *R* language and can operate on any of the Windows, Linux, and MacOS operating systems. The detailed guide for implementation is available in Supplementary Material S2. Testing the code for Scenario I on the simulated network of two layers with 100 nodes (see Supplementary Material) takes 243 s and requires 203 MB of RAM on a MacOS (version 13.4) and *R* version 4.3 (2023-06-16).

The use of the current EDNN, which is based on *multilayer perceptrons*, revealed two main limitations. Firstly, there is the challenge of scalability when dealing with exceedingly large networks. Secondly, selecting an appropriate distance measure between corresponding nodes within the embedding space lacks intuitive appeal. Both limitations can be addressed by integrating graph neural networks in subsequent versions of our pipeline.

## Data Availability

This data can be found here: 1) The data for Example 1 is available at: https://sites.broadinstitute.org/ccle. 2) The data for Example 2 belongs to the “Milieu Intérieur” study, Institut Pasteur, Paris, France.

## References

[B25] Bródka P. Chmiel A. Magnani M. Ragozini G. (2018). Quantifying layer similarity in multiplex networks: a systematic study. R. Soc. Open Sci. 5, 171747. 10.1098/rsos.171747 30224981 PMC6124071

[B1] Buphamalai P. Kokotovic T. Nagy V. Menche J. (2021). Network analysis reveals rare disease signatures across multiple levels of biological organization. Nat. Commun. 12, 6306. 10.1038/s41467-021-26674-1 34753928 PMC8578255

[B2] Corsello S. M. Nagari R. T. Spangler R. D. Rossen J. Kocak M. Bryan J. G. (2020). Discovering the anticancer potential of non-oncology drugs by systematic viability profiling. Nat. Cancer 1, 235–248. 10.1038/s43018-019-0018-6 32613204 PMC7328899

[B3] de la Fuente A. (2010). From ‘differential expression’ to ‘differential networking’ - identification of dysfunctional regulatory networks in diseases. Trends Genet. 26, 326–333. 10.1016/j.tig.2010.05.001 20570387

[B26] Fuller T. F. Ghazalpour A. Aten J. E. Drake T. A. Lusis A. J. Horvath S. (2007). Weighted gene coexpression network analysis strategies applied to mouse weight. Mamm. Genome 18, 463–472. 10.1007/s00335-007-9043-3 17668265 PMC1998880

[B4] Giri A. K. (2023). Exome-wide association study reveals 7 functional variants associated with ex-vivo drug response in acute myeloid leukaemia patients. bioRxiv. 10.1101/2023.08.02.23290523 PMC11969768 40186177

[B5] Hamilton W. L. (2017). Representation learning on graphs: methods and applications. arXiv [cs.SI]. 10.48550/arXiv.1709.05584

[B6] Hammoud Z. Kramer F. (2020). Multilayer networks: aspects, implementations, and application in biomedicine. Big Data Anal. 5, 2. 10.1186/s41044-020-00046-0

[B7] Howell A. Howell S. J. (2023). Tamoxifen evolution. Br. J. Cancer 128, 421–425. 10.1038/s41416-023-02158-5 36765172 PMC9938251

[B8] Ietswaart R. Gyori B. M. Bachman J. A. Sorger P. K. Churchman L. S. (2021). GeneWalk identifies relevant gene functions for a biological context using network representation learning. Genome Biol. 22, 55. 10.1186/s13059-021-02264-8 33526072 PMC7852222

[B9] Kuijjer M. L. Hsieh P. H. Quackenbush J. Glass K. (2019b). lionessR: single sample network inference in R. BMC Cancer 19, 1003. 10.1186/s12885-019-6235-7 31653243 PMC6815019

[B10] Kuijjer M. L. Tung M. G. Yuan G. Quackenbush J. Glass K. (2019a). Estimating sample-specific regulatory networks. iScience 14, 226–240. 10.1016/j.isci.2019.03.021 30981959 PMC6463816

[B24] Lichtblau Y. Zimmermann K. Haldemann B. Lenze D. Hummel M. Leser U. (2017). Comparative assessment of differential network analysis methods. Brief. Bioinform. 18, 837–850. 10.1093/bib/bbw061 27473063

[B11] Liu H. Liu K. Bodenner D. L. (2005). Estrogen receptor inhibits interleukin-6 gene expression by disruption of nuclear factor kappaB transactivation. Cytokine 31, 251–257. 10.1016/j.cyto.2004.12.008 16043358

[B12] Loscalzo J. (2017) Network medicine harvard university press.

[B13] Mahapatra S. Bhuyan R. Das J. Swarnkar T. (2021). Integrated multiplex network based approach for hub gene identification in oral cancer. Heliyon 7, e07418. 10.1016/j.heliyon.2021.e07418 34258466 PMC8258848

[B14] Matariek G. Teibo J. O. Elsamman K. Teibo T. K. A. Olatunji D. I. Matareek A. (2022). Tamoxifen: the past, present, and future of a previous orphan drug. EJMED 4, 1–10. 10.24018/ejmed.2022.4.3.1124

[B15] Melograna F. Li Z. Galazzo G. van Best N. Mommers M. Penders J. (2023). Edge and modular significance assessment in individual-specific networks. Sci. Rep. 13, 7868. 10.1038/s41598-023-34759-8 37188794 PMC10185658

[B16] Peng J. Zhou Y. Wang K. (2021). Multiplex gene and phenotype network to characterize shared genetic pathways of epilepsy and autism. Sci. Rep. 11, 952. 10.1038/s41598-020-78654-y 33441621 PMC7806931

[B17] Piasecka B. Duffy D. Urrutia A. Quach H. Patin E. Posseme C. (2018). Distinctive roles of age, sex, and genetics in shaping transcriptional variation of human immune responses to microbial challenges. Proc. Natl. Acad. Sci. U. S. A. 115, E488–E497. 10.1073/pnas.1714765115 29282317 PMC5776984

[B18] Shao N. Lu Z. Zhang Y. Wang M. Li W. Hu Z. (2015). Interleukin-8 upregulates integrin β3 expression and promotes estrogen receptor-negative breast cancer cell invasion by activating the PI3K/Akt/NF-κB pathway. Cancer Lett. 364, 165–172. 10.1016/j.canlet.2015.05.009 25979232

[B19] Thomas S. Rouilly V. Patin E. Alanio C. Dubois A. Delval C. (2015). The Milieu Intérieur study—an integrative approach for study of human immunological variance. Clin. Immunol. 157, 277–293. 10.1016/j.clim.2014.12.004 25562703

[B20] Van Dam S. Võsa U. van der Graaf A. Franke L. de Magalhães J. P. (2018). Gene co-expression analysis for functional classification and gene-disease predictions. Brief. Bioinform. 19, 575–592. 10.1093/bib/bbw139 28077403 PMC6054162

[B21] Yi L. Pimentel H. Bray N. L. Pachter L. (2018). Gene-level differential analysis at transcript-level resolution. Genome Biol. 19, 53. 10.1186/s13059-018-1419-z 29650040 PMC5896116

[B22] Yousefi B. Melograna F. Galazzo G. van Best N. Mommers M. Penders J. (2023). Capturing the dynamics of microbial interactions through individual-specific networks. Front. Microbiol. 14, 1170391. 10.3389/fmicb.2023.1170391 37256048 PMC10225591

[B23] Zheng D. Liwinski T. Elinav E. (2020). Interaction between microbiota and immunity in health and disease. Cell Res. 30, 492–506. 10.1038/s41422-020-0332-7 32433595 PMC7264227

