# Supplementary material for: PLEX.I: a tool to discover features in multiplex networks that reflect clinical variation
Source: Front Genet. 2023 Oct 19;14:1274637. doi: 10.3389/fgene.2023.1274637 (PMC10620964; doi:10.3389/fgene.2023.1274637)
Supplement: Supplementary file 1 [file DataSheet2.pdf]

Supplementary File 2

Data

In Scenario I, we used the gene expression data from lung cancer cell lines with their response to Tamoxifen in the PRISM dataset (Corsello et al., 2020), resulting in 80 samples. To classify the cell lines into the responder and nonresponder, we first sorted the IC50 values and detected the IC50 value at the point with the smallest derivative as the threshold. We then filtered out samples within a window 5% above and below this threshold resulting in 64 samples (27 responders and 37 nonresponders).

Results

The genes detected by PLEX.I are obtained using the default parameters set for the PLEX.I. Specifically, we used 2-dimensional embedding space and 50 repeats for the algorithm (see Supplementary File 1). The p-values obtained for each gene is adjusted for multiple testing using Bonferroni method and genes with final p-values < 0.01 are selected and listed in Table S1. The results of the gene in Table S1 are also considered for pathway overrepresentation analysis using Reactome Analysis Tools–Analyse gene list, and the resulting pathways with p-values < 0.01 are listed in Table S2.

We performed differentially expressed gene (DEG) analysis, with *alpha*=0.05 (using t-test) and $|\log_2(fold\ change)| > 1$, for response to tamoxifen and sex differences. We found genes listed in Table S3 for Scenario I, that have no intersection with the genes detected by PLEX.I, and found no genes for Scenario II.

Table S1. List of all genes detected by PLEX.I

Scenario	Gene set
Scenario I	GPR115, OR2A7, CAP2, SPNS2, MUC20, DSG2, MPZL3, TMC5, GSDMC, KPNA7, RASEF, DENND1C, TMEM61, ALDH3B2, OVOL2, VAMP8, TMPRSS2, ANXA9, SCNN1A, PTAFR, HSH2D, ZNF165, GOLM1, DAPP1, IKBIP, CD47, P2RY6, ARHGDIB, PROM2, STAP2, FAM83H, MYO6, PDXP, TMPRSS13, ITGB6, FXYD3, RBM47, DCLK2, ADAM8, IL1RN, ABCA12
Scenario II Escherichia coli stimulation	ADA, ARG2, ARHGDIB, ATG16L1, ATG7, ATM, BATF, BATF3, BAX, C3, C6, CAMP, CASP10, CCL20, CCL3, CD14, CD1D, CD209, CD22, CD274, CD276, CD40LG, CD46, CD48, CD79B, CD83, CD97, CEACAM1, CEACAM8, CFI, CLEC7A, CRADD, CSF1, CSF3R, CTLA4.TM, CTSC, CXCL12, CXCL2, CXCR2, DEFB1, DEFB103A, FCER1G, FCGR1A.B, GATA3, GNLY, GP1BB, GZMB, HLA.DMA, ICAM5, ICOS, IFI16, IFI35, IFNA2, IFNGR1, IKBKB, IL11RA, IL12A, IL16, IL17B, IL18RAP, IL19, IL1A, IL1B, IL1RN, IL21, IL23A, IL23R, IL26, IL28A.B, IL2RA, IL4R, IL6, IL6R, IL6ST, IRAK2, IRAK4, IRGM, KCNJ2, KIT, KLRG2, LEF1, LGALS3, LIF, LILRA6, LILRB5, LTBR, MAP4K1, MAP4K4, MASP2, MSR1, MX1, MYD88, NFIL3, NFKB2, NFKBIZ, NOS2, NOTCH1, PECAM1, PLA2G2E, PML, PPARG, PSMB7, PTPN22, RARRES3, RELA, S1PR1, SCTLA4, SELPLG, SERPING1, SLAMF7, SRC, TAGAP, TGFBR2, TLR2, TLR7, TLR8, TMEM173, TNFAIP3, TNFAIP6, TNFRSF13C, TNFRSF4, TNFSF12, TNFSF15, TNFSF4, TNFSF8, TP53, TRAF3, XCL1, ZEB1, GUSB, ABL1, CARD9, CD8B, CEACAM6, DPP4, FADD, HLA.C, ICAM4, IKZF2, KLRC3, KLRD1, KLRF1, MME, PRF1, PTK2, SELE, IRF3, ITGA4, RORC, TNFSF11, KLRC1
Scenario II Staphylococcus aureus stimulation	ABCB1, ATM, B3GAT1, C1S, CCL15, CCND3, CCR2, CD1D, CD209, CD22, CD244, CD4, CD45RB, CD46, CD86, CFD, CLEC7A, CMKLR1, CTSC, CXCR1, DEFB103A, EGR2, FCGR2B, GZMA, HAVCR2, HLA.DMA, HLA.DPA1, ICAM2, IFI35, IGF2R, IKZF1, IL12A, IL1R1, IL4, ILF3, IRGM, ITGAM, ITGAX, JAK1, KLRAP1, LAG3, LIF, LTA, MAP4K2, MASP2, MCL1, MYD88, NCR1, NFIL3, NOD2, NOTCH2, PLA2G2E, POU2F2, RARRES3, STAT2, STAT3, SYK, TIRAP, TLR7, TLR8, TNFRSF1B, TNFRSF9, TNFSF12, TRAF2, GAPDH, SDHA, CD24, CD8B, GZMK, ICAM4, KLRC2, RAG2, S100A8, ETS1, FYN, ZAP70, IKZF2, C14ORF166, CASP10, CD36, FCGR3A.B, MME, NFATC3, HPRT1, CASP2
Scenario II Staphylococcal enterotoxin B stimulation	IFNA2, IL10RA, IL1RL1, JAK1, LILRB3, MAPKAPK2, NFIL3, TCF4, TLR5, TYK2, PPIA, B3GAT1, BTK, CCBP2, CCR2, FCAR, HFE, IFNAR2, IKBKAP, IL17B, IL28A.B, NCAM1, RORC, TOLLIP, BATF3, BID, BST2, C2, CASP10, CCL13, CCL18, CCL19, CCL23, CCL24, CCL5, CCR5, CD163, CD1D, CD209, CD22, CD24, CD81, CDKN1A, CEBPB, CFD, CLEC5A, CLEC6A, CSF1R, CTSC, CXCL11, CYBB, EGR1, FAS, FCGR2B, HLA.DRB3, ICAM5, ICOS, IFI35, IL10, IL16, IL1R2, IL4, IL7R, IRF1, JAK2, KLRF1, LAIR1, LEF1, LGALS3, LIF, LILRB1, LTB4R2, MAP4K2, MARCO, MR1, MX1, MYD88, NFKB2, PLA2G2E, PRF1, PSMC2, RAF1, SELE, SIGIRR, SPP1, SRC, STAT3, TLR7, TNFAIP3, TNFAIP6, TNFRSF1B, ZEB1, HPRT1, BCL10, CD45R0, PSMB5, CD40LG, CTLA4, CXCL9, IL12B, LTA, TGFBI

Table S2. The result of pathway enrichment analysis entities with p-values < 0.01

Pathway identifier	Pathway name	Entities p.value	Entities FDR
R-HSA-6783783	Interleukin-10 signaling	1.621490956572602E-4	0.03128294696489853
R-HSA-9758274	Regulation of NF-kappa B signaling	0.0018681143694648794	0.03128294696489853
R-HSA-9013408	RHOG GTPase cycle	0.001955184185306158	0.03128294696489853
R-HSA-1660661	Sphingolipid de novo biosynthesis	0.002100218043785906	0.031503270656788596
R-HSA-9013407	RHOH GTPase cycle	0.0065052726161091146	0.0910738166255276
R-HSA-9020702	Interleukin-1 signaling	0.007258873107765185	0.09221464838286941

Table S3. List of all genes differentially expressed between responder and nonresponder cell lines to tamoxifen

Gene names
WDR63, NTNG1, SLC16A4, OLFML3, RGS1, LEMD1, TRIM58, OR2W3, SLC3A1, ZEB2, SP5, FAM3D, PRR16, CDX1, HAND1, HAVCR1, TLX3, DACT2, GNGT1, NPM2, GDF6, GPR20, RLN1, GRIN1, ST8SIA6, PLXDC2, APBB1IP, IGF2, IFFO1, C12orf56, NTS, SNORA31, PCDH20, FOXG1, C14orf23, SYT16, FBLN5, WFDC1, ALDH3A1, EVI2A, STAC2, KRTAP3-1, SKAP1, PHOSPHO1, AXIN2, RAB37, DSC3, SLC14A1, NETO1, KISS1R, PCP2, UNC13A, ZNF257, CXCL17, ZNF578, SNAP25, PCP4, TFF2, CSDC2, SYN1, BEX1, CDR1

References

Corsello,S.M. et al. (2020) Discovering the anticancer potential of non-oncology drugs by systematic viability profiling. Nature Cancer, 1, 235–248.
