# Supplementary material for: PLEX.I: a tool to discover features in multiplex networks that reflect clinical variation
Source: Front Genet. 2023 Oct 19;14:1274637. doi: 10.3389/fgene.2023.1274637 (PMC10620964; doi:10.3389/fgene.2023.1274637)
Supplement: Supplementary file 2 [file DataSheet1.PDF]

Supplementary File

1. Installation

Install the latest version from GitHub

```
devtools::install_github("behnam-yousefi/PLEXI/PLEXI")
library(PLEXI)
```

PLEXI will also install Keras for R, which need to be activated by installation of Miniconda. For this, according to the installation guidline of Keras (here):

```
library(keras)
install_keras()
```

This is required only once for the installation.

2. Apply on simulated networks

To test the `PLEXI` package, a toy example of multilayer network can be generated using the `network_gen()` function:

```
myNet = network_gen(n.nodes = 100, n.var.nodes = 5, n.var.nei = 90, noise.sd = .01)
```

The process is described as the following: 1. two fully connected networks with `n.nodes` number of matching nodes and uniform random edge weights is generated. 2. a number of `n.var.nodes` nodes are randomly selected to have different edge weights with `n.var.nei` number of nodes (`n.var.nei` $<$ `n.nodes` -1) between the two networks. 3. a set of random *Gaussian* noise with zero mean and sd = `noise.sd` is generated and added to all of the edge weights.

The generated multiplex network and the set of the randomly selected nodes are accessible by the following lines, respectively.

```
graph_data = myNet$data_graph
var_nodes = myNet$var_nodes
```

We then feed `graph_data` to the PLEXI pipeline specialized for a two-layer multiplex network (Scenario *I*), which is composed of two commands:

```
embeddingSpaceList = plexi_embedding_2layer(graph_data, train.rep = 50)
plexi_output = plexi_node_detection_2layer(embeddingSpaceList)
print(plexi_output$high_var_nodes_index)
```

the `plexi_embedding_2layer()` function represents all the nodes in a common embedding space (Section 2.1); and the `plexi_node_detection_2layer()` function calculates the node-pair distances and asignes a p-value to each node-pair (Section 2.2). This process is repeated `train.rep` times to improve the robustness. The source code available at Usage_Examples/network_generation_ex.R.

3. Usage Example 1: drug response

In this example, which is a showcase for Scenario *I*, we construct gene coexpression networks (GCNs) for drug responders and non-responders. To this end, we use the PRISM dataset (Corsello et al., 2020), which is a cell line-based drug screening dataset. To reduce the dimensionality, 2000 genes that are highly variant across all the cell lines are selected and reposited. The gene expression profile of lung cancer cell lines as `X` and a binary vector of their response to the *Tamoxifen* drug as `y` can be loaded accordingly:

```
data = readRDS("Data/GCN2Layer_data_lung_tamoxifen_2000genes.rds")
X = data[[1]]
y = data[[2]]
```

Next, we construct adjacency matrices of GCN for each condition,

```
adj_res = abs(cor(X[y=="res",]))
adj_nonres = abs(cor(X[y=="non_res",]))
diag(adj_res) = 0
diag(adj_nonres) = 0
```

and convert them into the `PLEXI` multiplex network format.

```
adj_list = list(adj_res, adj_nonres)
graph_data = as_plexi_graph(adj_list, outcome = c("res","non_res"))
```

Now we can call the PLEXI pipeline as in the previous example

```
embeddingSpaceList = plexi_embedding_2layer(graph_data, edge.threshold = .1,
  train.rep = 50,epochs = 20, batch.size = 10,
  random.walk = FALSE, null.perm = FALSE)
plexi_output = plexi_node_detection_2layer(embeddingSpaceList, p.adjust.method = "bonferroni")
Nodes = plexi_output$high_var_nodes
```

The source code available at Usage_Examples/drug_response_ex.R.

hints for large networks: * the random walk algorithm can be disabled by `random.walk = FALSE` to decrease the running time; * the network permutation and representation can be disabled by `null.perm = FALSE` to decrease the running time; * the calculated P-values can be adjusted by setting a method in the `p.adjust.method` argument.

4. Usage Example 2: application on individual specific networks

In this example we use the data of Milieu Interieur project (Thomas et al., 2015; Piasecka et al., 2018), where immune transcriptional profiles of bacterial-, fungal-, and viral- induced blood samples in an age- and sex-balanced cohort of 1,000 healthy individuals are generated. Here, the aim is to find genes whose neighborhood varition (from unstimulated to stimulated) is associated with sex. Following the PLEXI pipeline, we first construct a set of paired ISNs for the two conditions, i.e. before and after stimulation, using the *lionessR* R package (Kuijjer et al., 2019 a; Kuijjer et al., 2019 b). In each network, nodes and edge weights represent genes and the correlation of their expressions, respectively. The imputed ISNs are reposited in `"Usage_examples/Data/ISN_net.rds"`. We first read the ISN data and create the node list.

```
data = data.frame(readRDS("Data/ISN_BCG.rds"))
nodeList = t(sapply(rownames(data), function(x) strsplit(x,"_")[[1]]))
```

Next, we create the individual variable *data.frame* with three columns of *Individual IDs* (indecis), *stimulation condition*, and *sex* (F,M).

```
y = colnames(data)
y = data.frame(t(data.frame(strsplit(y, "_"))))
colnames(y) = c("ID", "Stim", "Sex")
```

Now that we have all the ISNs with their phenotypes, we can perform two types of analysis on the population level:

1- Aggregate ISNs (by averaging) into two groups (i.e. pre/post stimulation) and find genes with significant neighbourhood variation. This will be similar to *Usage Example 1* in the context of Scenario *I* (see above). We first obtain the two aggregated networks of pre- and post- stimulation;

```
data_agg = cbind(apply(data[,y$Stim=="Null"], 1, mean),
  apply(data[,y$Stim=="BCG"], 1, mean))
graph_data = cbind(nodeList, data_agg)
colnames(graph_data) = c("V1", "V2", "Null", "Stim")
```

and then perform the two-layer PLEXI pipeline.

```
embeddingSpaceList = plexi_embedding_2layer(graph_data, edge.threshold = .1,
  train.rep = 50, epochs = 25, batch.size = 10,
  random.walk = FALSE, null.perm = FALSE)
plexi_output = plexi_node_detection_2layer(embeddingSpaceList,
  p.adjust.method = "bonferroni", alpha = .01)
```

2- Project nodes of all the ISNs in the same embedding space and find significant genes, whose neighbourhood variants is associated with sex, in the context of Scenairio *II* (see above). In this analysis, the ISNs of pre- and post- stimulation should be paired. Therefore, for each individual-gene, we have two points in the embedding space: one correspond to pre-stimulation and the other correspond to post-stimulation. Calculating the distance between these pairs, we will have a matrix of distances of size $N_{individual} \times N_{gene}$.

To implement this, we use `plexi_embedding()` and `plexi_node_distance()` commands, respectively.

```
graph_data = cbind(nodeList, data)
embeddingSpaceList = plexi_embedding(graph_data, outcome = y$Stim, indv.index = y$ID,
  train.rep=50, walk.rep=10, epochs=10, batch.size=50,
  random.walk=FALSE)
Dist = plexi_node_distance(embeddingSpaceList)
```

Having the distance matrix, one can find association of any variable with them, for instance, here, we consider sex:

```
sex = y[duplicated(y$ID), "Sex"]
Pval = plexi_distance_test_isn(Dist, sex, p.adjust.method = "bonferroni")
```

The source code available at Usage_Examples/milieu_interior_ISN_sex_ex.R.

References

Corsello,S.M. et al. (2020) Discovering the anticancer potential of non-oncology drugs by systematic viability profiling. Nature Cancer, 1, 235–248.

Kuijjer,M.L. et al. (2019 a) Estimating Sample-Specific Regulatory Networks. iScience, 14, 226–240.

Kuijjer,M.L. et al. (2019 b) lionessR: single sample network inference in R. BMC Cancer, 19, 1003.

Piasecka,B. et al. (2018) Distinctive roles of age, sex, and genetics in shaping transcriptional variation of human immune responses to microbial challenges. Proc. Natl. Acad. Sci. U. S. A., 115, E488–E497.

Thomas,S. et al. (2015) The Milieu Intérieur study—an integrative approach for study of human immunological variance. Clin. Immunol., 157, 277–293.

Yousefi,B. et al. (2023) Capturing the dynamics of microbial interactions through individual-specific networks. Frontiers in Microbiology, 14, 1170391
